# Kinematics and Kinetics of Bulgarian-Bag-Overloaded Sprints in Young Athletes

**DOI:** 10.3390/life10110282

**Published:** 2020-11-15

**Authors:** Marco Duca, Athos Trecroci, Enrico Perri, Damiano Formenti, Giampietro Alberti

**Affiliations:** 1 Department of Biomedical Sciences for Health, University of Milan, 20122 Milan, Italy; marco.duca@unimi.it (M.D.); enrico.perri@unimi.it (E.P.); giampietro.alberti@unimi.it (G.A.); 2 Department of Biotechnology and Life Sciences, University of Insubria, 21100 Varese, Italy; damiano.formenti@uninsubria.it

**Keywords:** force–velocity relationship, speed training, youth, track and field

## Abstract

Background: Effective sprinting requires large acceleration capabilities. To accelerate, large amount of force must be produced and applied effectively. The use of different implements such as sleds and vests can increase the amount of force produced and alter sprinting effectiveness. We propose the use of increasing overload via the Bulgarian Bag (BB) as a means to modify athletes’ sprint and acutely increase force and power production. Methods: 24 young athletes performed three sprints over 20 m in three different conditions: unloaded (BW) and loaded with BB weighing 2.5% (BB2.5) and 5% (BB5) of the athlete’s body mass. Sprint times at 2.5, 5, 10, 15, and 20 m were acquired and used to compute the force–velocity relationship for the sprints. Maximal velocity (V0), peak force (F0), peak power (PP), and decrease in ratio of force (D_RF_) were computed. Results: the additional load caused a decrease in sprint times (*p* < 0.05) and V0 (*p* = 0.028), conversely no differences were found for F0 (*p* = 0.21), PP (*p* = 0.50), and D_RF_ (*p* = 0.83). Conclusions: Based on those findings, BB can be an alternative method to effectively overload sprint training toward improving sprinting performance.

## 1. Introduction

The acceleration phase is a key component of sprinting. In several sport activities (e.g., track and field and team sports), sprinting is not only substantial to reach maximal speed but also, and more importantly, to cover a given distance in the lowest time [1,2]. To effectively display this skill, large forward acceleration is needed. Acceleration is related to the capability to produce and to apply in the horizontal direction large amount of force into the ground at increasing speed throughout the acceleration phase [1,3].

The total mechanical capacity to produce horizontal force during sprint is described by its linear inverse relationship with velocity (F–v), while the horizontal power is shown to be in a parabolic relationship with speed (P–v) [4]. It is known that, albeit F–v relationships obtained on a detached muscle fiber or regarding mono-articular movements are described by Hill’s hyperbolic equation, the F–v relationships obtained are linear when considering multi-articular movements such as squatting or running [1,5]. These relationships characterize the external mechanical limit of the entire neuromuscular system during a specific multi-articular action. Moreover, the slope of F–v relationship defines the mechanical F–v profile, which is the individual relationship between strength and speed capabilities.

During sprinting, the mechanical properties obtained by F–v and P–v relationships correspond to a complex integration between the different mechanisms involved in total strength production. These mechanisms comprise the mechanical characteristics of the muscle, morphological factors, and individual neural mechanisms [6]. Moreover, acceleration requires force production in two directions (horizontal and vertical) with F–v and P–v relationships portraying the ability to effectively apply force into the ground [3,4]. The mechanical effectiveness (ME) during sprint is quantified at each step by the ratio of force (RF) between the force applied in horizontal direction and the total ground reaction force (GRF). Considering the whole acceleration phase, ME is described by the decrease of the RF (D_RF_) as speed increases. D_RF_ is unrelated to the total force applied and describes the athlete’s ability to maintain the GRF vector directed forward despite the increasing speed [7].

Resisted sprint training is a common training modality in both individual and team sports [8,9,10]. The resistance can be applied to the athlete using a sled, to be towed or pushed [11], or wearing a weight, in the form of a weighted vest [8] or a Bulgarian Bag (BB). This added resistance alters the athlete’s position during sprinting and constrains the athlete to apply larger forces into the ground compared to unresisted sprinting [12]. These two factors contribute to the effectiveness of resisted sprinting at improving sprint times [8,9,10]. Sprint kinematics with different overloads was in fact investigated by Cronin et al. [12]. The authors compared the acute effects of weighted vest and sled towing equal to 15–20% of body mass on sprint kinematics over 30 m in 20 young track athletes and rugby union players. The two conditions determined similar decrements in step length and step frequency while the duration of the stance phase increased. Additionally, it was shown that sled towing increased trunk forward lean angle while weighted vest decreased it [12]. Effects on ME were also assessed during sled towing [13,14], while no information concerning weighted vests is present. The type of resistance and its application point appear to be crucial in determining the position of the segments of the body during the acceleration phase [11]. Sleds are usually towed by athletes with a belt insisting on the hips [13] and more rarely using a shoulder vest harness [15]. While both hip and shoulder attachments lead to similar alterations in GRF compared to unresisted sprinting, they differ regarding knee joint kinematics, with the shoulder attachment displaying larger knee flexion [15]. While vests apply their masses on the top of the shoulders when standing [12], during acceleration athletes lean forward, thus the weight of the vest is more evenly distributed on the trunk.

The BB is an implement imposing its weight at a more cranial point, thus possibly modifying sprinting mechanics. Indeed, imposing its load on the base of the neck can result in an alteration of the athlete’s posture and alter sprint kinematics, kinetics, and ME. Unfortunately, to the authors’ knowledge, the literature provides no information on this matter. Therefore, the aim of this study was to analyze the effects of BBs of increasing mass—equal to 2.5% (BB2.5) and 5% (BB5) of the athlete’s body mass—on sprinting kinematics, kinetics, and ME compared to the unloaded condition (BW).

## 2. Materials and Methods

### 2.1. Participants

Twenty-four young athletes (14 females and 10 males; age range: 13.4–15.2 years) were recruited to take part in the study. They had been involved in track and field for 2 years, practicing 3 times a week for 1.5 h and participating in one competition event per week. Due to injury or personal reasons, five subjects (3 females and 2 males) did not complete the testing protocols and were excluded from analysis. As a result, a total number of 11 females and 8 males completed the entire experimental protocol, and their anthropometrical characteristics are shown in Table 1. The experimental protocol was approved by the Institutional Review Board of the University of Milan (2/12) in compliance with the Helsinki declaration. All subjects and their guardian or parents were informed of the risks and benefits of the investigation prior to obtaining signed consent.

### 2.2. Procedures

A cross-over design was employed to investigate the acute effects of overload via BB on sprint performance. Testing was conducted outdoors in favorable environmental conditions (no wind, no rain) on a synthetic athletic track (from 5.00 to 6.00 p.m.) over three days interspersed by 72 h. On the first testing day, a familiarization session was provided to get the participants accustomed with all experimental procedures. In this session, anthropometrical measurements including height, sitting height, and body mass were measured using a stadiometer and a portable scale to the nearest 1.0 cm and 0.1 kg, respectively. Given these variables, the corresponding maturity offset (MO) was also calculated by the equation of Mirwald et al. [16] to monitor potential difference in maturity status. Then, all participants performed 3 sprints over 20 m [17] with 2 min of passive recovery between bouts and initiated the sprint after a countdown [18].

**Figure 1:**
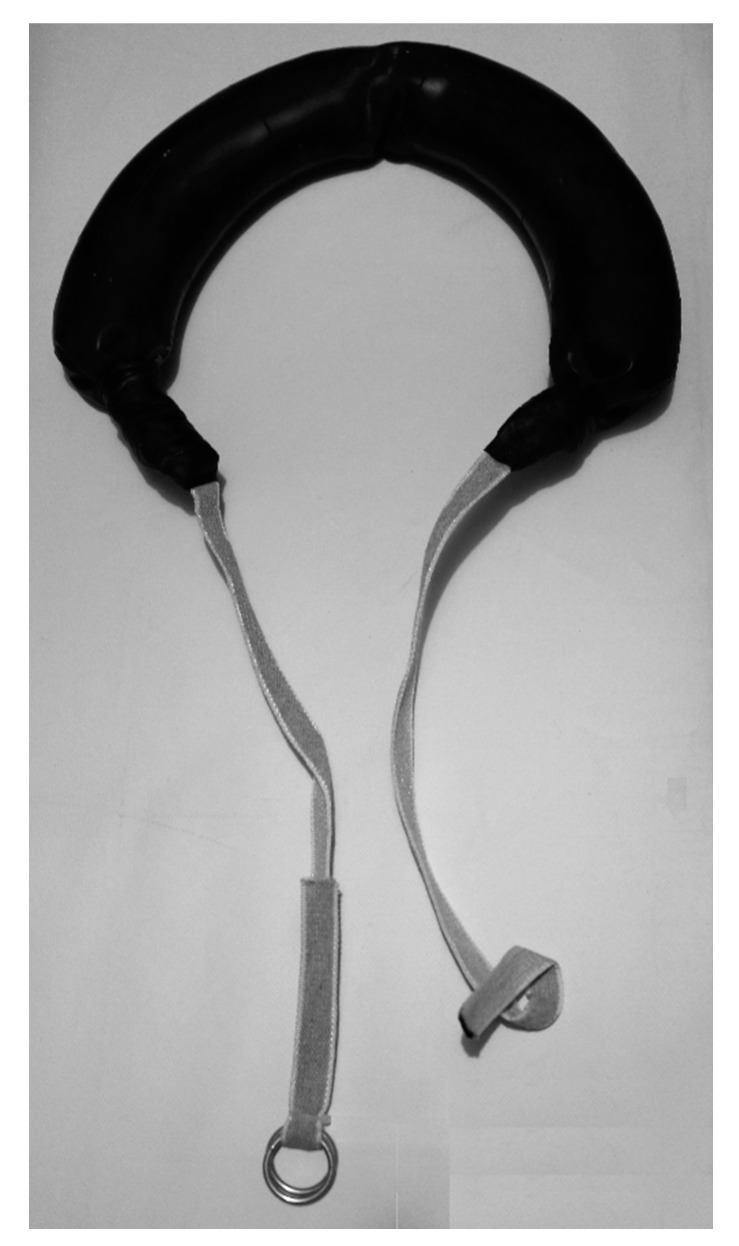
Bulgarian Bag (BB) used for overloading athletes during sprinting. The bag was placed on the athletes’ neck. The strings at each end of the BB were crossed over their chests and then firmly secured between the athletes’ scapulae via the pair of metal rings connected to the strings.

During the second and third testing sessions, all the participants underwent a sprint protocol consisting of three different experimental conditions: with no overload (BW), with BB equal to 2.5% of the athlete’s body mass (BB2.5), and with BB equal to 5% of the athlete’s body mass (BB5) (Figure 1). The tests were repeated for reliability purposes.

Each athlete performed the sprints in the three conditions in one of the six possible different sequences used to account for the possible potentiation effect provided by the overloaded condition on the subsequent one [19]. The participants performed 3 sprints of 20 m in each of the three conditions (BW, BB2.5, and BB5). The rest between sets and repetitions was set at 5 and 2 min, respectively.

**Figure 2:**
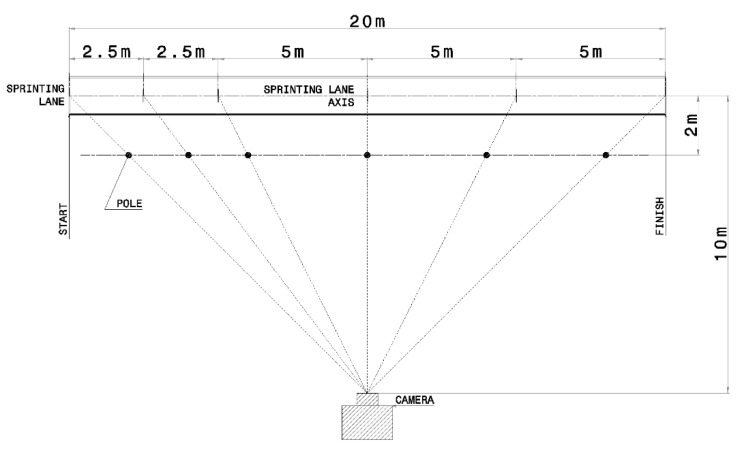
Layout of the on-field video acquisition system. Six vertical poles were placed 2 m from the sprinting lane axis and corresponding to start line, 2.5, 5, 10, 15, and 20 m. The camera was set 10 m apart from the sprint lane axis.

All sprints were filmed using a high-speed digital camera (FDR1000V, Sony, Tokyo, Japan). The camera filming speed was set at 240 fps, with a resolution of 720 p [20]. Six vertical poles were placed between the sprinting lane axis and the camera lens, 2 m offset from the sprinting lane axis, on the imaginary lines drawn from camera lens and start line, 2.5, 5, 10, 15, and 20 m points on the sprinting lane axis (Figure 2). This setting allowed to measure the running time of the athletes by an inexpensive and feasible approach previously presented in the literature [20].

### 2.3. Data Analysis

For each athlete, the best out of three trials for each condition was analyzed using a field-based method [7]. This analysis consisted of an inverse dynamic analysis of athlete’s center of mass motion. Partial times at 2.5 m (T2.5, s), 5 m (T5, s), 10 m (T10, s), 15 m (T15, s), and 20 m (T20, s) were captured using a video analysis software (Kinovea^®^ 0.8.15). The stopwatch provided by the software was started when the athlete’s sternum–clavicular joint reached the vertical pole placed near the start line; the same anatomical landmark was used to stop the chronometer at the 2.5, 5 m10, 15, and 20 m marks.

From those 5 split times [21], it was possible to calculate the mean speeds for each interval (d) section as follows:

(1) $$\bar{v}=\frac{\Delta d}{\Delta t}=\frac{d}{t_{1}-t_{0}}$$

During sprint with maximal acceleration, the relationship between horizontal velocity ($v_{H}$) and time (t) follows a mono-exponential function for both trained sprinters and recreational practitioners [22,23,24]:

(2) $$v_{H}\left(t\right)=v_{H_{max}}\cdot \left(1-e^{-t/\tau }\right)$$

Replacing $v_{H}\left(t\right)$ with $\bar{v}$(t), where (t) is the mean time for each section for which $\bar{v}$ was computed, and where $v_{H_{max}}$ was estimated as $\bar{v}$ in the last section (15–20 m), it is possible to estimate τ for $\bar{v}$(t):

(3) $$\tau =-\frac{t}{\ln\left(1-\frac{\bar{v}\left(t\right)}{\bar{v}{}_{15-20m}}\right)}$$

Afterward, the mean ($\bar{\tau }$) from the four τ values was calculated. $\bar{\tau }$ was employed for computing instantaneous speed $v_{H}\left(t\right)$ and acceleration $a_{H}\left(t\right)$, when take-off occurred during first, second, third steps, at 10 and at 20 m. (t) were acquired by video analysis:

(4) $$v_{H}\left(t\right)=\bar{v}{}_{15-20m}\cdot \left(1-e^{-t/\bar{\tau }}\right)$$

(5) $$a_{H}\left(t\right)=\left(\frac{\bar{v}{}_{15-20m}}{\bar{\tau }}\right)\cdot e^{-t/\bar{\tau }}$$

Using the fundamental law of dynamics on the horizontal axis, net ground antero-posterior reaction force ($F_{H})$ applied by the body’s centre of mass (CM) can be modeled in function of time as:

(6) $$F_{H}\left(t\right)=m\cdot a_{H}\left(t\right)+F_{aero}\left(t\right)$$

where m is the sum of athlete’s body mass and the mass of the BB used. $F_{aero}\left(t\right)$ was the aerodinamic friction to be overcome during running, equal to air speed squared respective to the runner, multiplied by a k factor:

(7) $$F_{aero}\left(t\right)=k\cdot {\left(v_{H}\left(t\right)-v_{w}\right)}^{2}$$

where $v_{w}$ was wind speed and k the aerodynamic friction coefficient. k can be estimated, as proposed by Arsac and Locatelli [25], from air density values (ρ in kg·m^−3^), sprinter’s frontal area (Af, in m^2^), and drag coefficient (Cd = 0.9) [26]:

(8) k=0.5·ρ·Af·Cd

(9) $$\rho =\rho_{0}\cdot \frac{Pb}{760}\cdot \frac{273}{273+T{}^{\circ}}$$

(10) $$Af=\left(0.2025\cdot h^{0.725}\cdot m^{0.425}\right)\cdot 0.266$$

where $\rho_{0}$ = 1.293 kg·m^−1^ is ρ at 760 Torr and 273 K, Pb the barometric pressure (in Torr), T° the air temperature (in °C) and h the athlete’s height (in m).

On the vertical axis, the sprinter’s CM, during the acceleration phase, rises from a crouched position (either using or not using starting blocks), to the standing running position. A small vertical acceleration is present as the rising of CM is achieved over the whole acceleration phase and not in a single step [27]. Therefore, mean vertical acceleration at each step during acceleration can be considered as null. Then, applying the fundamental law of dynamics in the vertical direction, the mean GRF applied by the CM ($F_{V}$) can be estimated as a function of time as the sum of athlete’s body mass and the mass of the BB (g = 9.81 m·s^−2^) [28]:

(11) $$F_{V}\left(t\right)=m\cdot g$$

The F–v relationship is determined using mean values of $F_{H}$ and $v_{H}$ and applying the least squares method [3,29]. The maximal theoretical velocity (V0, m·s^−1^) and the maximal theoretical force (F0, N) are extrapolated by the F–v relationship, as the relationship intercepts with the vertical axis and the horizontal axis, respectively. Dividing F0 by the athlete’s body mass, it was possible to assess the relative maximal theoretical force (F0_rel_, N·kg^−1^). The peak power of the relationship (PP, W) was calculated via the previously validated formula [4,30]:

(12) $$\mathrm{PP}=\frac{F0\cdot V0}{4}$$

Additionally, dividing PP by the athlete’s body mass, the relative peak power (PP_rel,_ W·kg^−1^) was computed.

In the literature, it was proposed that the ME of force application during running could be quantified over each support phase or step by the ratio (RF in %) of $F_{H}$ to the corresponding total resultant GRF ($F_{res}$, in N) [1]:

(13) $$RF=\frac{F_{H}}{\sqrt{F_{H}{}^{2}+F_{V}{}^{2}}}\cdot 100$$

Then, an index of technical application of force (D_RF_)—describing RF decrease as speed increases—was computed. As speed raises, RF tends to zero due to the absence of a positive resultant acceleration in the horizontal direction. This computation exploits mean values at each step starting from the second step to the last step over 20 m. A higher D_RF_ value (namely a flat RF–v relationship) means a larger maintenance of *RF* as velocity increases [1].

### 2.4. Statistical Analysis

The normality distribution of each variable was examined using the Shapiro–Wilk’s normality test. Inter-test reliability for sprint times between the two testing occasions was quantified by a two-way mixed intraclass correlation coefficient (ICC) for average measurements (ICC type 3, k) and interpreted as poor (<0.40), fair (0.40–0.60), good (0.60–0.75), and excellent (0.75–1.00) [31]. Standard error of measurement (*SEM*) was computed for sprint times using the following equation:

(14) $$SEM=SD\cdot \sqrt{1-ICC}$$

where *SD* is the standard deviation of the sample.

One-way repeated-measures analyses of variance (ANOVA RM) was used to assess differences among the three overload conditions (BW, BB2.5, and BB5) for the dependent variables. In the case of a significant effect, a Bonferroni post hoc test was applied for multiple comparisons. The significance level was set at 0.05.

To determine the practical significance of the overload condition on the dependent variables, effect sizes (ESs) were computed using Cohen’s f and interpreted as large (≥0.5), medium (0.25–0.5), small (0.1–0.25), or null (<0.1). ESs for pairwise comparisons were computed using Cohen’s d and interpreted as large (≥0.8), medium (0.5–0.8), small (0.2–0.5), or null (<0.2).

All statistical analyses were performed using SPSS v.21.0 (IBM Corp., Armonk, NY, USA) and a customized Excel sheet (Microsoft, Redmond, WA, USA).

## 3. Results

Test–retest reliability measured by ICC was excellent for sprint times under all conditions (BW = 0.820, BB2.5 = 0.854, BB5 = 0.849). SEM for test day sprint times was 0.05 s.

**Figure 3:**
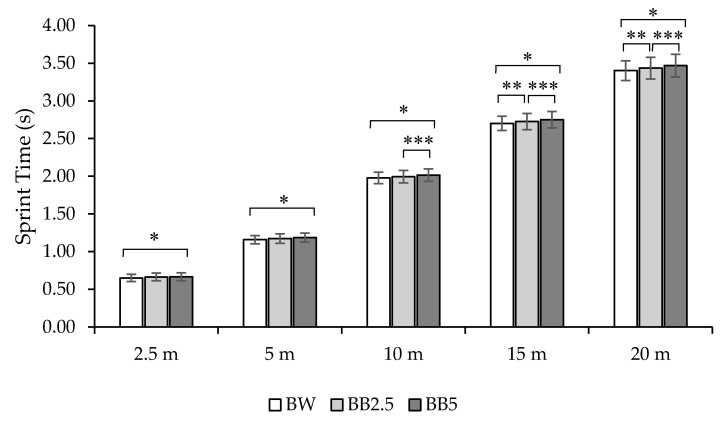
Bar graph showing sprint times at 2.5, 5, 10, 15, and 20 m in the three different overloading conditions. BW = no overload; BB2.5 = Bulgarian Bag overload equal to 2.5% of the athlete’s body mass; BB5 = Bulgarian Bag overload equal to 5% of the athlete’s body mass; * = significant difference at *p* < 0.05 between BW and BB5 conditions; ** = significant difference at *p* < 0.05 between BW and BB5 conditions; *** = significant difference at *p* < 0.05 level between BB2.5 and BB5 conditions.

For all sprint times (Figure 3), ANOVA RM detected significant differences: T2.5m (F(2,36) = 4.23, *p* = 0.02), T5m (F(2,36) = 7.29, *p* = 0.002), T10m (F(2,36) = 8.77, *p* < 0.001), T15m (F(2,36) = 12.31, *p* < 0.001), T20m (F(2,36) = 13.35, *p* = 0.0005). Post hoc tests results for sprint times are displayed in Table 2.

Descriptive data and ANOVA RM for the dependent variables computed from the F–v relationship are shown in Table 3.

Post hoc analysis was applied only to V0, significant difference was found only for BW vs. BB5 conditions (T(18) = 2.54, *p* = 0.02). No significant difference was found for BW vs. BB2.5 (T(18) = 1.99, *p* = 0.06) or for BB2.5 vs. BB5 (T(18) = 1.13, *p* = 0.28). ESs for all variables are shown in Table 4.

## 4. Discussion

The main finding of this study was that, albeit a significant reduction in sprint times, the use of overload via BB did not deteriorate force production and mechanical effectiveness when compared to the unloaded condition. To the authors’ knowledge, this is the first study investigating the effects of overloaded sprinting in young athletes using the BB.

Times to cover all distances increased in both BB2.5 and BB5 conditions over BW condition. These findings are consistent with other studies involving sprinting with increasing overloads [13,32,33]. In these studies, sprinters performed sled towing and, as the overload increased, the time to cover 20 and 30 m distances underwent a significant deterioration [13,32]. The same increase in sprint times was presented for weighted vests in a recent review [33]. This deterioration in sprint times is reflected by a reduction in V0. As a matter of fact, a decrease in sprinting velocities was reported for the increasing overloads [13,32]. The same effect was tangible for the subjects involved in our study.

Kinetic analysis derived from the F–v relationship revealed medium effects for the total amount of force produced and relative force production. A small effect was present when comparing BB5 to BB2.5 and BB2.5 to the BW condition. The increase in overload enabled a larger force to be produced by the athletes. This finding is consistent with other studies considering sled towing [13,34]. Kawamori et al. [34] found no significant increase over bodyweight condition for lighter loads, and a sled loaded with 30% of the athlete’s body mass was necessary to elicit a greater horizontal force output. Similar results were found by Monte et al. [13], who investigated adult male sprinters performing sled towing with overloads equal to 15%, 20%, 30%, and 40% of the athlete’s body mass. Compared to the unloaded condition, the force produced in the 15% condition was larger, as was the force produced in the 20% condition compared to that in the 15% condition. No further increase in force production was found, as it stabilized for 30% and 40% conditions. Conversely, a recent review showed that weighted vests, with overloads ranging from 5% to 40% of the athlete’s body mass, decreased force production by 6–7% and power production by 11–14% [33].

In our investigation, peak power did not display any alteration with increasing overloads. In the case of sled towing, Monte et al. [13] noticed a range of optimal overloads for power production, corresponding to overload of 15% to 20%. This discrepancy in the results could be due to the lighter overloads imposed on the young athletes involved in the present study. It is possible that both force production and power production would still have increased with heavier overloads. In contrast with the other investigations, our data did not show power production capabilities to be impaired by increasing overloads.

No differences in ME were present when comparing the three different loading conditions. To the authors’ knowledge, this is the first study reporting the effects of increasing overloads on ME. It is possible to suggest that this lack of differences could be due to the light overloads used in the investigation. The BBs used were not heavy enough to determine a harmful alteration in sprint, that could result in compromising the athletes’ ME.

The contrasting results could be due to the different point at which the BB imposed its weight on the athlete compared to weighted vests, similar to what was presented for the harness attachment point in the case of sled towing [15]. Harness attachment on the shoulder resulted in altered kinematics of the trunk, hips, and knee and a lesser force and power production compared to that for the hip attachment. Similar differences could be present when comparing the BBs, that can be tightly fit around the athletes’ shoulders and therefore imposing all their weights on the most cranial portion of the trunk, to weighted vests that can fit more loosely around the athletes’ waist. The difference may occur especially during the first steps of the acceleration phase, when the athletes are leaning forward, and the weighted vest imposes most of its weight on the lower portion of the athlete’s back. Therefore, the BB can be used to apply the resistance in a different way compared to weighted vests or sleds, exploiting the possible benefits on force production presented in this study, potentially leading to improvement in sprinting performance.

The main limitation of the present study refers to the athletes’ young age and relative inexperience with sprint training, that likely contributed to the high variability in sprint performance data.

In conclusion, overload via BB did not impair force and power production capabilities and maintained unaltered ME. Thus, we can recommend to coaches to prescribe BB-resisted sprint sessions over short distances (~20 m) with full recovery. BB can be an alternative method to effectively overload sprint training toward improving sprinting performance. Of note, the BB should be selected according to the individual athlete’s body mass and tightly fit around their trunk, so as to avoid any bouncing of the implement on their necks and shoulders.

## Figures and Tables

**Table 1:** Participants’ anthropometric characteristics (sample size = 19).

	Mean ± SD
Stature (m)	1.60 ± 0.06
Body mass (kg)	53.6 ± 8.9
Maturity Offset (years)	1.1 ± 1.2

SD = standard deviation.

**Table 2:** Bonferroni post hoc comparisons for the sprints completed in different conditions.

	BW–BB2.5		BW–BB5		BB2.5–BB5	
	T	*p*	T	*p*	T	*p*
T2.5 (s)	−2.09	0.051	−2.97	0.008 *	−0.75	0.466
T5 (s)	−2.07	0.053	−4.85	0.0001 *	−1.37	0.188
T10 (s)	−1.66	0.115	−5.59	0.00003 *	−2.13	0.047 *
T15 (s)	−2.14	0.046 *	−5.83	0.00002 *	−2.52	0.021 *
T20 (s)	−2.47	0.024 *	−5.31	0.00005 *	−2.64	0.017 *

BW = no overload; BB2.5 = Bulgarian Bag overload equal to 2.5% of the athlete’s body mass; BB5 = Bulgarian Bag overload equal to 5% of the athlete’s body mass; T2.5 = time over 2.5 m; T5 = time over 5 m; T10 = time over 10 m; T15 = time over 15 m; T20 = time over 20 m; * = significant difference at level of *p* < 0.05.

**Table 3:** Descriptive statistics and ANOVA RM results for the dependent variables.

	BW	BB2.5	BB5	ANOVA RM	
	mean ± SD	mean ± SD	mean ± SD	F_2,36_	*p*
V0 (m·s^−1^)	7.33 ± 0.44	7.26 ± 0.46	7.19 ± 0.51	3.95	0.028 *
F0 (N)	525.3 ± 96.2	541.8 ± 94.4	551.1 ± 130.5	1.65	0.21
F0_rel_ (N·kg^−1^)	9.92 ± 0.91	10.28 ± 1.16	10.37 ± 1.54	1.55	0.23
PP (W)	966.1 ± 206.1	984.3 ± 193	990.9 ± 237.8	0.71	0.50
PP_rel_ (W·kg^−1^)	18.18 ± 2.08	18.61 ± 2.14	18.6 ± 2.54	0.72	0.50
D_RF_	−0.078 ± 0.009	−0.079 ± 0.01	−0.079 ± 0.012	0.18	0.83

BW = no overload; BB2.5 = Bulgarian Bag overload equal to 2.5% of the athlete’s body mass; BB5 = Bulgarian Bag overload equal to 5% of the athlete’s body mass; V0 = maximal theoretical velocity; F0 = maximal theoretical force; F0_rel_ = maximal theoretical force relative to body mass; PP = peak power; PP_rel_ = peak power relative to body mass; D_RF_ = decrease in ratio of force; * = significant difference at level of *p* < 0.05.

**Table 4:** Effect sizes for the dependent variables.

			BW–BB2.5		BW–BB5		BB2.5–BB5	
	Cohen’s *f*	Inference	Cohen’s *d*	Inference	Cohen’s *d*	Inference	Cohen’s *d*	Inference
T2.5 (s)	0.48	large	0.25	small	0.08	null	0.33	small
T5 (s)	0.64	large	0.25	small	0.20	null	0.45	small
T10 (s)	0.70	large	0.20	small	0.26	small	0.47	small
T15 (s)	0.83	large	0.23	small	0.25	small	0.48	small
T20 (s)	0.86	large	0.23	small	0.23	small	0.46	small
V0 (m·s^−1^)	0.47	medium	−0.17	null	−0.13	null	−0.30	small
F0 (N)	0.3	medium	0.15	null	0.09	null	0.24	small
F0_rel_ (N·kg^−1^)	0.29	medium	0.29	small	0.08	null	0.37	small
PP (W)	0.2	small	0.09	null	0.03	null	0.12	null
PP_rel_ (W·kg^−1^)	0.2	small	0.19	null	0.00	null	0.19	null
D_RF_	0.1	small	−0.1	null	0.05	null	−0.05	null

BW = no overload; BB2.5 = Bulgarian Bag overload equal to 2.5% of the athlete’s body mass; BB5 = Bulgarian Bag overload equal to 5% of the athlete’s body mass; V0 = maximal theoretical velocity; F0 = maximal theoretical force; F0_rel_ = maximal theoretical force relative to body mass; PP = peak power; PP_rel_ = peak power relative to body mass; D_RF_ = decrease in ratio of force.
